# Microalgal Phenolics: Systematic Review with a Focus on Methodological Assessment and Meta-Analysis

**DOI:** 10.3390/md22100460

**Published:** 2024-10-07

**Authors:** Vasilis Andriopoulos, Michael Kornaros

**Affiliations:** 1 Laboratory of Biochemical Engineering & Environmental Technology (LBEET), Department of Chemical Engineering, University of Patras, 26504 Patras, Greece; billandri@upatras.gr; 2 Institute of Circular Economy and Environment (ICEE), University of Patras’ Research and Development Center, 26504 Patras, Greece

**Keywords:** HPLC, LC-MS, interference, heavy metal, shikimate pathway, phenylpropanoid pathway, benzoic acid, caffeic acid, flavonoids, adsorption

## Abstract

A critical review and analysis of the literature relevant to the phenolic content of eucaryotic microalgae was performed. Several issues were identified and discussed. In summary, the main problems with the reporting on the phenolic content of microalgae are the following: (1) despite its usefulness in the determination of phenolic content in plant samples, the Folin–Ciocalteu assay is non-suitable for microalgal research due to the high presence of interfering compounds in microalgal extracts such as chlorophyll and its derivatives in organic extracts and free aromatic amino acids or nucleotides in aqueous extracts; (2) while there is chromatographic evidence for the presence of simple phenolic acids in most microalgal clades, the lack of critical enzymes of phenolic biosynthesis in most microalgae, as well as the high variability of phenolic profiles even in the same genus, require more extensive research before conclusions are drawn; (3) the accumulation and metabolism of external phenolics by microalgae has been almost universally neglected in studies focusing on the phenolic content of microalgae, even when natural seawater or complex organic media are used in the cultivation process. Despite these issues, the literature focusing on the bioremediation of waste streams rich in phenolics through microalgae demonstrates the ability of those organisms to adsorb, internalize, and in many cases oxidize or transform a wide range of phenolic compounds, even at very high concentrations. Simple phenolics found in waste streams, such as olive mill waste, have been shown to enhance the antioxidant activity and various bioactivities of microalgal extracts, while complex biotransformation products of phenolics have also been characterized. In conclusion, the de novo biosynthesis of phenolic compounds via eucaryotic microalgae requires further investigation with better designed experiments and suitable analytical methods, while the response of microalgae to phenolic compounds in their growth medium is of great practical interest, both in terms of waste treatment and for the production of functional foods, cosmetics, and pharmaceuticals.

## 1. Introduction

Phenolic compounds are a diverse group of chemical substances characterized by the presence of one or more hydroxyl groups attached to an aromatic ring [1]. They have played an enormous role in the history of our planet, specifically with the evolutionary invention of lignin, which allowed plants to dominate the land and form the ecosystem with which we are familiar [2]. While lignin is a primary component of wood, phenolics are generally regarded as secondary metabolites, meaning they are not directly involved in the growth, development, or reproduction of plants but play crucial roles in the alleviation of stress conditions and defense against pathogens, UV radiation, and herbivores [3]. For example, research has demonstrated that phenolic compounds like flavonoids, tannins, and lignins are crucial for plants’ ability to withstand various biotic and abiotic stresses [4]. Other phenolic compounds are important in soil; for example, as chelators that allow roots to absorb limiting nutrients [5]. Overall, phenolics play an enormous role in the life of plants, and consequently in the life of humans. 

Ancient civilizations harnessed the benefits of these bioactive molecules, laying the foundation for traditional medicine systems. The Egyptians, for instance, used plant extracts rich in phenolics for embalming and as antiseptics [6,7]. In traditional Chinese medicine, herbs such as green tea (*Camellia sinensis*) and ginkgo (*Ginkgo biloba*), known for their high phenolic content, have been employed for their therapeutic effects [8,9]. Similarly, in the Mediterranean, the use of olive oil (*Olea europaea*) and wine, both rich in phenolic compounds, dates back thousands of years and continues to be integral to cultural practices and health regimens [10]. Over the centuries, botanists and biologists have systematically studied phenolics, elucidating their roles in plant defense, growth, and reproduction, as well as their diverse structural variety and potent antioxidant properties. 

Apart from plants, macroalgae, like kelp, also contain phenolic compounds, with similar functions to those of terrestrial plants [11,12]. These organisms are the trees of the sea, covering large portions of the sea bottom proximate to the ocean coastline [13]. Some of them produce large amounts of phenolics like phlorotannins, which have antioxidant, antiproliferative, or antidiabetic properties, and have thus attracted interest in the search for novel drugs [12]. In recent years, unicellular algae, or microalgae, have also been in the spotlight due to their phenolic content. Microalgae are omnipresent in aquatic systems around the globe and have attracted a lot of interest as sources of food, fuel, and bioactive compounds. So far, the cost of microalgal cultivation is high, which is detrimental to the upscaling of their commercial production. The co-production of high-value products, such as antioxidants, along with bulk products such as protein or lipids is a way to improve the economic viability of microalgal production [14,15,16]. Therefore, the production of phenolics from microalgae could be of great financial importance. 

Unfortunately, there are several issues with the methodology and reporting of the phenolic content of microalgae, including the use of unsuitable analytical methods and possible contamination from external sources of phenolics. The current article aims to present the findings on the phenolic content of microalgae while focusing on the potential pitfalls of the experimental methodology used. Also, a meta-analysis is attempted, meaning a statistical analysis of the available data that will assist in the interpretation of the existing literature findings. As described by Borenstein et al., “Meta-analysis refers to the statistical synthesis of results from a series of studies” [17]. Initially, the term was used in clinical studies of single variables or effects; however, it has been extended to virtually all research areas [9,18,19,20] and multivariable studies [21]. Overall, this approach should provide a clear view of the existing data on microalgal phenolics, while identifying underlying problems that need to be resolved.

Briefly, Section 2 provides an overview of the phenolic content of microalgae measured with the Folin–Ciocalteu assay, while the interference of non-phenolic compounds such as pigments, amino acids, and nucleotides, and the potential stimulation of phenolics biosynthesis by heavy metals, are discussed. Section 3 focuses on the chromatographic data of microalgal phenolics, with an extensive discussion, backed by statistical analysis, of potential problems in the methodology that was applied, as well as the paradoxical absence of key metabolic pathways for phenolic biosynthesis in most microalgae. 

## 2. Estimation of Total Phenolic Content by the Folin Ciocalteu Assay in Microalgae

The most common method for measuring the phenolic content in various fields, such as botany [22], food analysis [23], and oenology [24], is the Folin–Ciocalteu (FC) assay. This is a quick and sensitive method with a limited number of steps and reagents, and has thus been widely adopted. Initially, a similar technique was developed by Folin and Denis for the quantification of tryptophan and other aromatic amino acids [25]. The method was later adjusted by Folin, Ciocalteu, and Denis for the detection of phenolic acids, and later modified by Singleton [26,27,28]. Therefore, the fact that the method was prone to interference from amino acids was known since its creation. Decades later, more interfering molecules were identified, including ascorbic acid and purines [29]. Below, a historical overview of the use of the FC assay in microalgal research and an analysis of the potential problems with the use of that method are presented.

### 2.1. Historical Perspective

The FC assay was occasionally used by phycologists studying macroalgae and microalgae until the 2000s, but it became mainstream in microalgal research with papers such as those of Li et al. and Goiris et al., in 2007 and 2012, respectively [30,31], around the time when the number of publications in the field increased exponentially [32]. Li et al. sequentially extracted microalgae and cyanobacteria from different phyla with polar and non-polar solvents and assessed the total phenolic content, which was as high as ~60.4 mg/g of dry weight (DW) for cyanobacteria and ~19 mg/g DW for microalgae. This phenolic content is lower than that of the richest phenolic sources, such as olives or raisins, but comparable to that of herbs like oregano and basil [33,34]. However, the authors observed that there was no correlation between the total phenolic content determined by the FC assay (TPC) and the antioxidant capacity of the extracts, the opposite to what is observed in plant extracts.

In the following years, the interest in the phenolic content of microalgae increased, with the application of various extraction techniques and combinations of the FC assay with different analytical methods being reported. A significant, but until recently overlooked, paper was that of Cha et al., who combined pressurized fluid extraction with an online HPLC ABTS^•+^ analysis [35]. The results clearly indicated that under mild extraction conditions (ambient pressure and low–medium temperatures) the antioxidant activity was mainly attributed to chlorophyll and its derivatives, while the contribution of polar compounds was only significant at high pressures and temperatures. The same year (2010), another group also used highly pressurized liquid extraction and, additionally, HPLC/ESI-MS to show the existence of simple phenolic acids, at submicrogram per gram DW levels, in the eucaryotic green microalgae *Spongiochloris spongiosa*, as well as some cyanobacteria and red macroalgae [36]. For *S. spongiosa,* the TPC of the extract was ~3 mg/g dry extract (DE), around 600 times higher than the total chromatographically identified phenolics. 

Later, various groups, like those of Custódio et al. and Goiris et al., correlated the TPC of polar and non-polar microalgal extracts with different in vitro activities [31,37]. Custódio et al. found a non-zero TPC only in methanolic extracts of *Nannochloropsis oculata*, *Tetraselmis chuii*, *Chlorella minutissima,* and *Rhodomonas salina*. However, this was not reflected in the radical scavenging activity and AchE inhibition, which were much higher in hexane extracts, with zero TPC. Iron chelating ability, however, was comparable between the methanolic and hexane extracts. The authors attributed the activity of the hexane extracts to unsaturated fatty acids, sterols, and tocopherols, which they identified with GC-MS. On the other hand, Goiris et al. studied hydroethanolic, hexane, and aqueous extracts of 32 different microalgae, and reported the highest TPC in ethanol–water extracts. Also, they correlated Ferric ion reducing antioxidant power (FRAP), ABTS radical scavenging activity, and the inhibition of the AAPH-induced oxidation of linoleic acid with both TPC and the carotenoid content. Both classes of compounds were significant, while the overall fit of the linear models was low. 

During the following decade, much more research on the phenolic content of microalgae by the FC assay was published, either presenting TPC along with antioxidant assays or in combination with the chromatographic results. Many researchers focused specifically on HPLC or LC-MS identification and/or the quantification of phenolic compounds. In 2016, chlorophyll was identified as an interfering compound in an FC assay of sunflower oil [38]. In 2022, the interference of chlorophyll was also recognized in microalgal extracts by two different groups [39,40]. Ben Hamouda et al. extracted *Haematococcus pluvialis* with various solvents and found a linear interference of both Chla and Chlb in the FC assay. After removing all pigments from ethanolic extracts (95% EtOH), they observed a decrease of ~50% in TPC and concluded that the remaining activity could be attributed to phenolics or other polar compounds [39]. Andriopoulos et al. correlated the chlorophyll content of methanolic extracts of various marine microalgae with TPC. LC-MS analysis showed the absence of phenolic compounds from the extracts with the highest TPC [40]. To our knowledge, chlorophyll interference in the FC assay has not been addressed by others since then. 

### 2.2. Chlorophyll Interference

**Figure 1:**
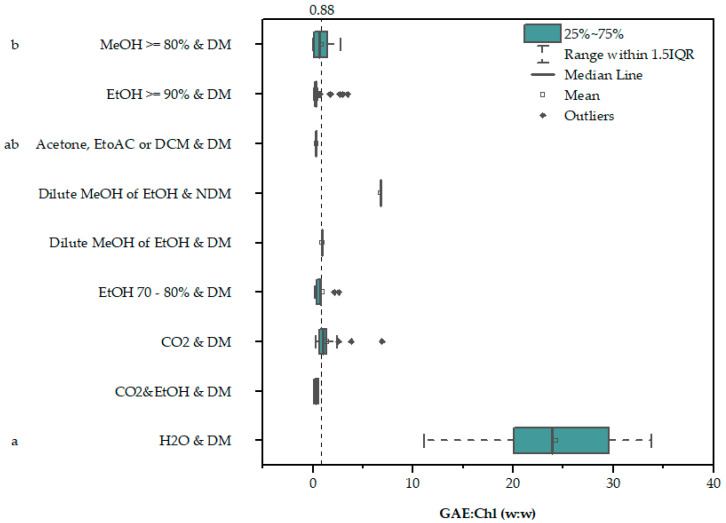
Gallic acid equivalents (GAEs) to chlorophyll ratio for various solvents and growth medium compositions (defined or not). Data were collected and processed according to the Section 5, subsections “Data Collection” and “Estimation of chlorophyll interference”. All data are available in Supplementary Data—GAE-Chl_ratio. The dotted vertical line indicates the interference observed by Ben Hamouda et al., ~0.88 GAE:Chl w:w (detailed explanation provided in the Section 5, subsection “Estimation of chlorophyll interference”). Significant differences (*p* < 0.05) are indicated by a compact letter display. The TPC of the data points on the left of the interference line can be attributed solely to the chlorophyll content. Boxes contain values between the first and third quartiles, while the minimum and maximum values are indicated with vertical bars at the end of lines that extend from the boxes. Values more than 1.5 times greater or lower than the interquartile range (the difference between the first and third quartiles) shown for the boxes are considered outliers.

Figure 1 [39,40,41,42,43,44,45,46,47,48,49,50,51,52,53,54] and Figure S1 [39,40,41,42,43,44,45,46,47,48,49,50,51,52,53,54,55] present the mass ratio of gallic acid equivalents to chlorophyll (GAE:Chl w:w) for different solvents and growth media compositions, as well as for the different references used for its estimation. The interference observed by Ben Hamouda et al. (detailed explanation provided in the Section 5, subsection “Estimation of chlorophyll interference”) is also plotted for reference. Chlorophyll interference is potentially significant for polar organic solvents, but also for supercritical CO_2_ extraction.

**Figure 2:**
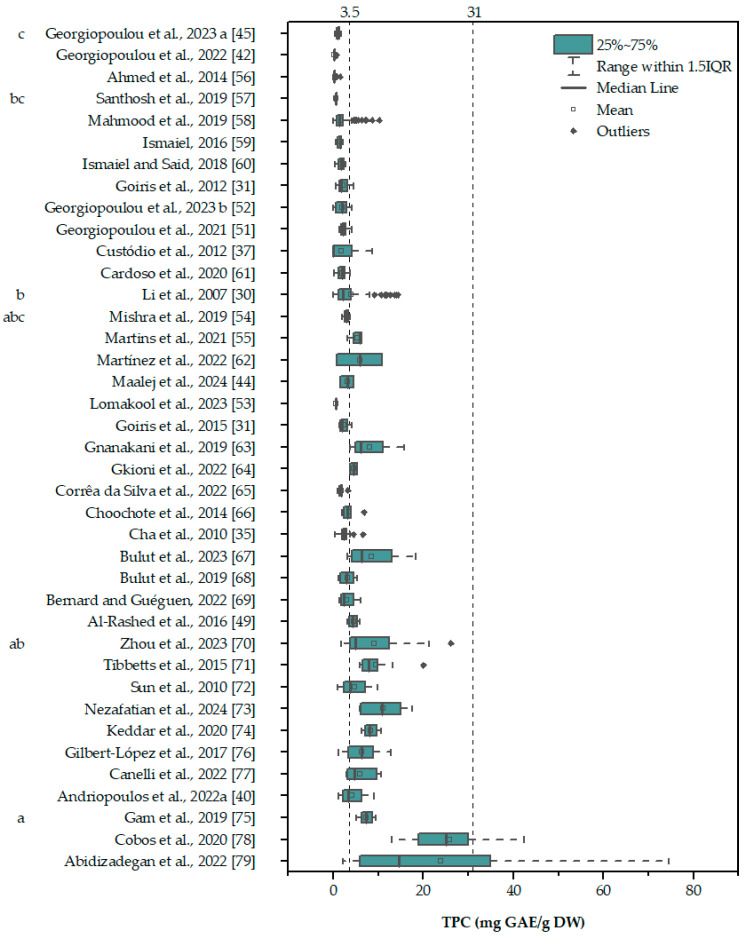
Reported TPC in terms of the dry weight of the biomass for the different references used in this study. The dotted vertical lines indicate the minimum and maximum chlorophyll interference expected assuming a chlorophyll content ~35 mg Chl/g DW (detailed explanation provided in the Section 5, subsection “Estimation of chlorophyll interference”). The total phenolic content below the upper limit of the interference (31 mg GAE/g DW) could be attributed to the presence of chlorophyll rather than the presence of phenolic compounds. Significant differences (*p* < 0.05) are indicated by a compact letter display. Boxes contain values between the first and third quartiles, while the minimum and maximum values are indicated with vertical bars at the end of the lines that extend from the boxes. Values that are more or less than 1.5 times the interquartile range (the difference between the first and third quartiles) are considered outliers.

The data for water are from the only reference in the current study that provided the chlorophyll content of the aqueous extracts [41]. In that case, it was obvious that water-soluble compounds were responsible for the registered phenolic content, since a large GAE:Chl ratio indicates that the FC signal cannot be explained solely by the presence of chlorophyll. It is also interesting to note that the only data point in this figure without a defined medium is well outside the borders of chlorophyll interference, which will be discussed later. In order to estimate the possible chlorophyll interference in the data that do not provide the chlorophyll content, an approximate mean value of ~3.5% Chl per gram was used to estimate a range for the potential chlorophyll interference, between 3.5 and 31 mg GAE/g DW (a detailed explanation is provided in the Section 5, subsection “Estimation of chlorophyll interference”), and plotted against the literature data (Figure 2 [30,31,35,37,40,42,43,44,45,49,51,52,53,54,55,56,57,58,59,60,61,62,63,64,65,66,67,68,69,70,71,72,73,74,75,76,77,78,79]).

Given that the chlorophyll content can be as high as 5% [50,80], it is evident that chlorophyll interference poses a significant problem for the estimation of phenolic content using the FC method. 

### 2.3. Other Interfering Molecules

Apart from chlorophyll, there are other interfering molecules that are often neglected. Kilari and Balakrishnan detected mainly amino acids and purines in ethanolic (90%) extracts of *Chlorochromonas danica*, while no phenolic acids or flavonoids were found [81]. Guanine, one of the major components that was found, is particularly reactive towards the FC reagent, with a response of ~0.34 mg GAE/mg Guanine [29].

By comparison, the response of aromatic amino acids is in the range 0.28–0.41 mg GAE/g [29]. The DNA and RNA content of microalgae is high, requiring their removal from certain microalgal products intended for food [82]. The leakage of purines from denatured DNA or RNA during extraction could have a significant impact on their reactivity towards FC, while free amino acids can represent up to 20% of the total protein [83], which makes their significant presence in aqueous extracts very likely. Other water-soluble interfering molecules like ascorbic acid have been studied more extensively, probably due to their higher presence in plant samples [84]. However, the particularly high content of microalgae in nucleic acids and amino acids makes the FC method problematic even in the case of aqueous extracts.

### 2.4. Phenolic Response of Microalgae to Heavy Metal Stress

A scenario that deserves to be examined independently is the phenolic response of microalgae to heavy metal stress. Plants have evolved various biological mechanisms to cope with the presence of heavy metals in their environment [85]. These mechanisms include the production of metal-binding proteins like metallothioneins and phytochelatins, which sequester and detoxify heavy metals by binding them. Phenolics also act as metal chelators and are used by microorganisms and plants to either neutralize toxic metals in the environment or to increase the bioavailability of iron and other micronutrients [86]. Microalgae are known to withstand high concentrations of heavy metals [87] and thus the mechanism of their response to this stress has been studied.

Glutathione and ascorbic acid are both reactive towards the FC assay and have been found to play a role in microalgae’s response to heavy metal stress. Strejckova et al. examined the response of *Scenedesmus* to different levels of copper, cadmium, and nickel [88]. They observed the increased oxidation of glutathione and ascorbic acid with increasing heavy metal concentrations, as well as an increasing concentration of phytochelatin, an oligomer of glutathione, in the cells. TPC increased in the presence of heavy metals, especially at intermediate concentrations. However, TPC levels were high even in the control, and it is not clear what solvent was used for extraction and if the phenolic content was presented in terms of biomass or extract weight. Additionally, they measured different simple phenolics, specifically rosmarinic acid and its precursors, phenylpyruvic acid, phenylacetic acid, 4 hydroxyphenylpyruvic acid, and 3,4 dihydroxyphenylacetic acid, with liquid chromatography coupled with mass spectrometry [88]. Elleuch et al. examined the effect of increasing zinc concentration on the antioxidant response of *Dunaliella* [89]. They extracted fresh biomass with a neutral buffer containing EDTA and measured the total phenolic, flavonoid (TFC), and tannin (TCT) content, as well as the activities of various antioxidant enzymes. TPC, TFC, and TCT increased until a certain zinc concentration, with the same being observed for guaiacol peroxidase activity and glutathione content. NMR identified carboxylic, carbonyl, and phosphate groups as the main participants in zinc biosorption. Bernard and Guéguen studied the effects of five different carbon sources on the phenolic content of *Euglena gracilis* [69]. Using HPLC and quadrupole time-of-flight (QTOF) mass spectrometry, they did not find any of the nine phenolic standards (gallic acid, catechin, chlorogenic acid, caffeic acid, p-coumaric acid, ferulic acid, naringenin, quercetin, and kaempferol) used in the study in methanolic or ethanolic extracts, while the highest TPC was observed when glutathione was used as the carbon source. However, they characterized the peaks in unidentified compounds in terms of their elemental composition and found an increase in molecules containing nitrogen and sulfur during the log-phase, when glutathione was used as a C-source. This evidence paints a blurred picture, where the possible de novo synthesis of phenolics in response to heavy metal stress might be masked by high concentrations of other antioxidants. 

## 3. Qualitative and Quantitative Analysis of Phenolics in Microalgae with Liquid Chromatography (LC) Methods

**Figure 3:**
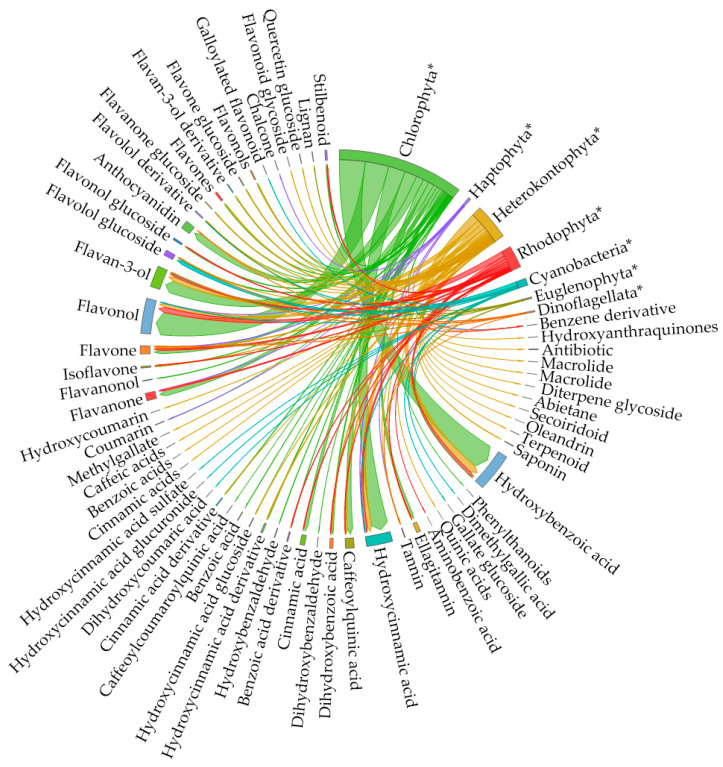
Chord diagram of major types of phenolic compounds (and some non-phenolic compounds like polyketides and isoprenoids) identified chromatographically in different microalgal Phyla. Microalgal phyla are distinguished from identified compounds with an asterisk at the end (*). The arc length of every item on the diagram is proportional to the number of times the item is present in the data (all data are provided in the Supplementary Data, Sheet “Figures S3 and S6_Data”).

While, based on the previous analysis, the phenolic content of microalgae measured by the FC assay needs reconsideration, phenolic compounds such as hydroxybenzoic acids, hydroxycinnamic acids, flavonols, flavanones, coumarins, and a range of glycosylation products have been identified in different microalgal phyla with chromatographic methods coupled with UV/Vis or MS detectors (Figure 3 [44,53,58,61,62,65,67,68,70,75,81,88,90,91,92,93,94,95,96,97,98,99,100,101], Table S1 [40,44,58,61,65,67,68,69,75,90,91,92,93,94,96,98,99,100,101,102,103]). This evidence is hard to dismiss, since an increasing number of researchers report both quantitative and qualitative results. Below, an overview of the relevant literature is presented, while potential problems are discussed and analyzed.

### 3.1. Overview of Chromatographic Data

**Figure 4:**
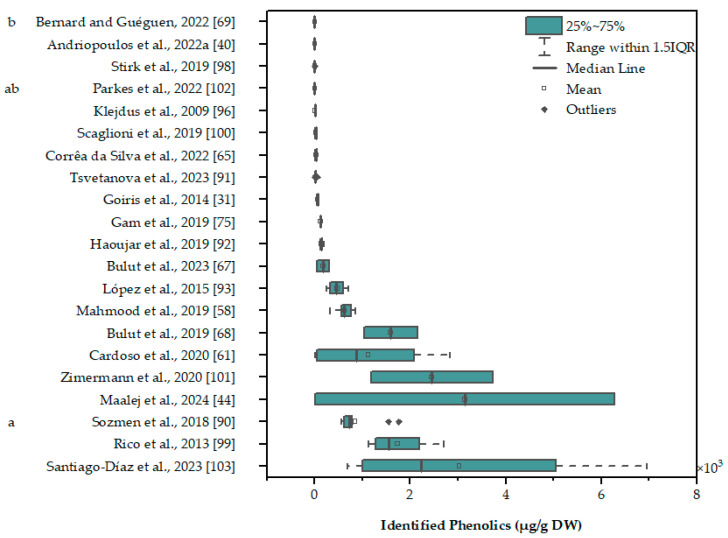
Total identified phenolics in different references used in this article. Significant differences (*p* < 0.05) are indicated by compact letter displays. Boxes contain values between the first and third quartiles, while the minimum and maximum values are indicated by the vertical bars at the end of lines that extend from the boxes. Outliers are considered values more than 1.5 times greater or smaller than the interquartile range (the difference between the first and third quartiles).

As early as 2001, Miranda et al. identified simple phenolic acids, namely salicylic, trans-cinnamic, synaptic, chlorogenic, chimic, and caffeic acids, in *Chlorella vulgaris* using gas-chromatography coupled with a flame ionization detector [97]. Unfortunately, only the abstract of this article is available online and thus the specific derivatization process used by the authors cannot be assessed. Later, Klejdus et al. used solid-phase/supercritical-fluid extraction and reverse-phase HPLC coupled with ESI-MS to detect selected phenolics (benzoic acid derivatives, hydroxy-benzaldehydes, and cinnamic acid derivatives) in the green microalgae *Spongiochloris spongiosa* and some cyanobacteria [96]. Additionally, they quantified the standard phenolics, with p-hydroxybenzoic acid and p-hydroxybenzaldehyde being the most abundant in *S. spongiosa*, at levels of ~2.2–2.7 μg/g DW, while the total identified phenolics were ~5.7 μg/g DW (Figure 4 [40,44,58,61,65,67,68,69,75,90,91,92,93,94,96,98,99,100,101,102,103]). Shortly after, Onofrejová et al. confirmed those results using similar methods [36]. Goiris et al. performed an extensive study of the phenolic content of microalgae from different phyla (UHPLC-MS/MS), using only distilled water in the preparation of growth media and treating the extracts with ion exchange chromatography to remove pigments and other non-relevant compounds [31]. They found only submicrogram levels of phenolic acids and flavonoids, while phloroglucinol, a simple phenol produced by brown algae, was detected in much higher concentrations, ~40–90 μg/g DW.

Others focused on the effects of different biomass pre-treatments, extraction methods, or solvents on the phenolic content of microalgal extracts. For example, Tsvetanova et al. found a much higher phenolic content in Soxhlet ethanolic extracts of *Porphyridium cruentum* and *Scenedesmus* sp. than in supercritical CO_2_–ethanol extracts [91]. Martínez et al. detected several bioactive compounds in *Nannochloropsis gaditana* that was either non-processed or treated with steam explosion [62]. Additionally, they subjected both the treated and non-treated biomasses to enzymatic digestion. Among the compounds identified with UPLC-QTOF, caffeic acid glycoside was found only in the digested untreated biomass, while quercetin glycoside was found only in non-treated biomass. Mahmood et al. optimized the extraction of *Chlorella vulgaris* with deep eutectic solvents (DES) [58]. In the optimized DES, gallic acid content was as high as 670 μg/g DW compared to the 130 and 30 μg/g DW found for ethyl acetate and water, respectively, while the change in total phenolics was not that pronounced. 

Some research groups studied the effects of various growth parameters on the phenolic content of microalgae rather than screening different species or evaluating extraction protocols. For example, Rico et al. examined the effects of iron and copper concentrations on the phenolic profile and content of the diatom *Phaeodactylum tricornutum* [99]. They observed a significant change with increasing metal concentrations, particularly in catechin levels when cells were exposed to copper. Interestingly, gallic acid was only found in the iron treatment. The authors also reported the phenolic content of the cell exudates, which was lower than the control in the iron treatment, but more than two-fold that of the control in the highest copper treatment. The same group also examined the effect of the same conditions in *Dunaliella tertiolecta* [93]. This time, they observed a decrease in the biomass phenolic content in the presence of metals, while the exudate of the iron treatment had double the phenolic content of the other treatments. Later, they also studied the effect of *P. tricornutum* exudate on the reduction in ferric iron (Fe^3+^) to ferrous iron (Fe^2+^) and found an increase in the reduction rate due to catechin and sinapic acid [104]. In the same paper, they also reported the phenolic content of the natural seawater used in the preparation of the growth substrate, which was lower than that of exudates. However, approximately half of the phenolic compounds found in biomass extracts were also present in the seawater, with the most abundant being catechin and sinapic acid.

Sozmen et al. also studied the effects of growth conditions on the phenolic content of microalgae [90]. *Chlorella miniata* was cultivated in Bristol protease broth, which contains proteolytic animal products, and exposed to different temperatures and light intensities. They reported comparatively high levels of most examined phenolics—for example, up to ~70 μg/g DW for trans-cinammic acid and up to ~35 μg/g DW for the flavanol myricetin—while other compounds were found in lower levels—for example, catechin (<2.5 μg/g DW). On the other hand, the concentrations of salicylic and caffeic acids were much higher, with the former reaching up to ~650 μg/g DW and the latter exceeding 1 mg/g DW. Interestingly, caffeic acid concentration was very high up to a light intensity of 112 μmol ph m^−2^ s^−1^, above which a sudden drop in levels to < 20 μg/g DW was observed. Another study where a very high content of single identified phenolics was reported is that of Zimermannet al., where the catechin content of the thermophilic red microalgae *Galdieria sulfuraria* was as high as 3.6 mg/g DW in shake flask cultures [101]. Interestingly, catechin was absent in bioreactor cultures; ellagic acid was the dominant phenolic, which was absent in the case of shake flask cultures. That study also used a complex organic medium of animal origin, whey permeate, which was not analyzed for phenolics, and the cultivation mode was heterotrophic (no light). The authors suggested that the different dissolved oxygen and pH conditions between the bioreactor and shake flask cultures may have influenced the biosynthesis of phenolics by *Galdieria sulphuraria*. A thermotolerant strain of *Scenedesmus* also presented high concentrations of benzoic acid derivatives, cinnamic acid derivatives, and flavonols, as high as ~654, ~352, and ~845, respectively [68]. On the other hand, Maalej et al. found a high concentration of phenolics in *Scenedesmus*, but only when it was cultivated in medium containing olive mill waste (OMW) [44]. The total content of identified phenolics was ~350 higher in the treatment with OMW, while the main compounds were coumaric acid and hydroxytyrosol. 

In contrast to the high phenolic concentrations reported in some of the studies mentioned before, others did not find any phenolic compounds in microalgal samples (Figure 4). Parkes et al. did not detect any of the reference phenolics in the diatom *Stauroneis sp.* or the green microalgae *Tetraselmis chuii* using HPLC-UV, and used the same method to measure different phenolics in blueberry fruit extracts and basil leaf extracts [102]. Andriopoulos et al. also did not detect phenolics in extracts of *Chlorella minutissima*, *Dunaliella salina*, *Tisochrysis lutea*, *Isochrysis galbana,* or *Nannochloropsis oculata* using LC-MS [40]. Bernard and Guéguen examined the phenolics in *Euglena gracilis* cultivated under different carbon sources using HPLC-Q-TOF-MS, but could not detect any of the nine standard compounds used in any sample [69]. On the other hand, in another study, *E. gracilis* growth, chlorophyll content, and lipid accumulation were enhanced in the presence of p-coumaric acid and syringic acid [105], both used as standards by Bernard and Guéguen [69]. The studies of Parkes et al., Andriopoulos et al., and Bernard and Guéguen used different extraction methods, specifically mechanical homogenization with methanol, mixing with methanol, and ultrasound-assisted extraction with ethanol, respectively. A common factor between them, however, is the washing of the biomass prior to the extraction, as well as the application of a post-treatment to the extract. 

In summary, a range of simple phenolic compounds has been identified and quantified in various microalgae. The results, however, are highly diverse and estimating the gravity of the many parameters involved is challenging. An attempt is made to explain some of the observed results and assess possible pitfalls in their presentation and interpretation.

### 3.2. Incomplete Phenylpropanoid Metabolic Pathway

**Figure 5:**
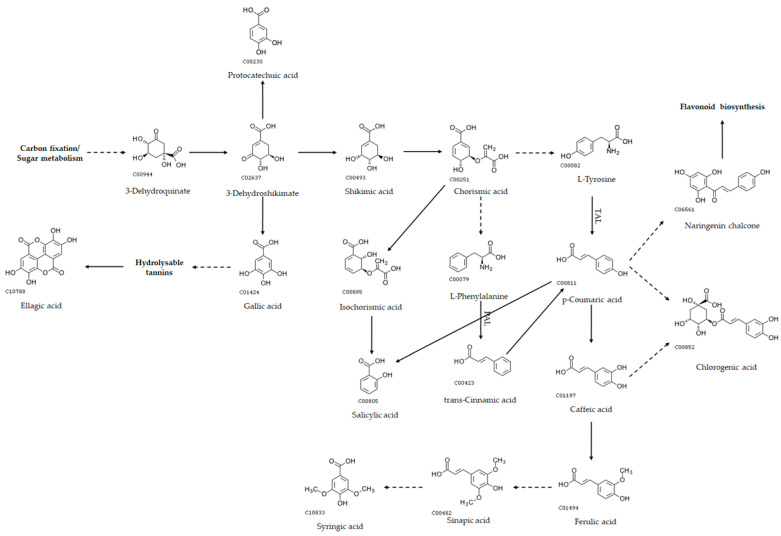
Some key aspects of the biosynthesis of phenolics in plants. Phenylalanine ammonia lyase (PAL) and tyrosine ammonia lyase (TAL) are shown. Multiple reaction steps are indicated with dotted lines. Compounds were retrieved from KEGG (https://www.genome.jp/kegg/, accessed on 27 September 2024).

The biosynthesis of phenolics in plants starts with the Shikimate pathway, which ends with the production of the aromatic amino acids tyrosine, phenylalanine, and tryptophan [106] (Figure 5). 

**Figure 6:**
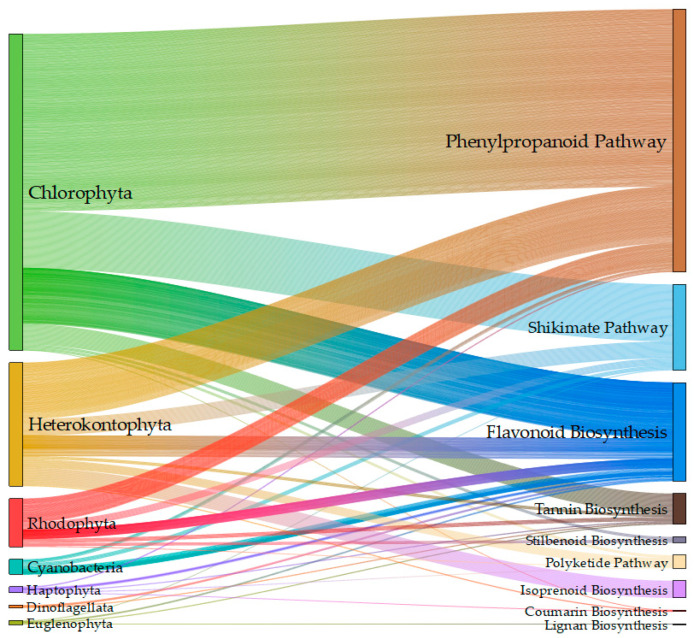
Sankey plot of the distribution of identified compounds in different metabolic pathways and microalgal Phyla. The height of bars on the left is proportional to the number of times a compound was identified in a specific Phylum. The height of bars on the right is proportional to the number of times a compound originating from a given metabolic pathway was identified.

Dehydroshikimate, an intermediate of the pathway, is a precursor to the benzoic acids gallic and protocatechuic [107]. Gallic acid is an important precursor in the biosynthesis of hydrolysable tannins, which, upon hydrolysis, produce polyphenols such as ellagic acid [108]. Tyrosol and hydroxytyrosol, abundant in olives, are probably synthesized from derivatives of tyrosine [109], and are thus also indirect products of the Shikimate pathway. Phenylalanine or tyrosine initiate the Phenylpropanoid pathway, with their deamination to cinnamic acid and coumaric acid by phenylalanine ammonia lyase (PAL) and tyrosine ammonia lyase (TAL), respectively [110]. Cinnamic acid derivatives such as caffeic acid and ferulic acid are precursors to hydroxybenzoic acids such as salicylic, sinapic, and syringic acids. Both products of the Shikimate pathway and the Phenylpropanoid pathway have been detected in microalgae (Figure 6 [44,53,58,61,62,65,67,68,70,75,81,88,90,91,92,93,94,95,96,97,98,99,100,101]), with the positive detection of gallic and protocatechuic acids being reported in at least 12 different articles, and chlorogenic, syringic, vannilic, caffeic, and coumaric acids in 12–17 articles (Table S1). Amino acid synthesis is very active in microalgae, and thus Shikimate pathway products can be expected. The presence of Phenylpropanoid pathway products, however, is hard to explain in some cases, since the first enzyme of the pathway (PAL or TAL) is missing from most eucaryotic microalgae, as discussed in the recent review of Del Mondo et al. [110].

**Figure 7:**
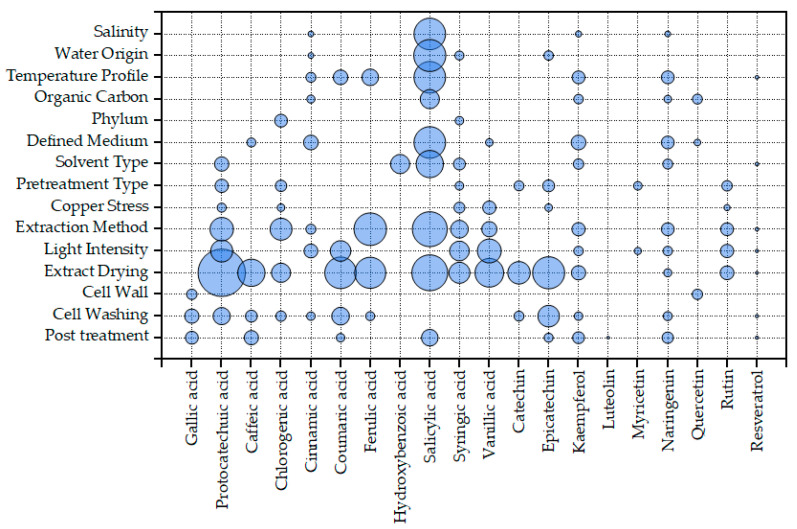
Bubble chart of Kruskal–Wallis test results for the effect of various parameters on the biomass concentration in individual phenolics. Bubble diameter is proportional to the effect size, Epsilon squared (a detailed explanation is provided in the Section 5, subsection “Statistical analysis”). Only results deemed significant using the Benjamani–Hochberg procedure (detailed explanation provided in the Section 5, subsection “Statistical analysis”) are presented. Molecules are sorted by their biosynthesis pathway (from left to right; Shikimate pathway, starting with gallic acid; Phenylpropanoid pathway, staring with caffeic acid; Flavonoid pathway; starting with catechin; and Stilbenoid pathway, represented by resveratrol).

There is growing evidence for the importance of benzoic and salicylic acids as signaling molecules or hormones in *Chlorella* and other green microalgae [111,112,113]. In land plants, salicylic acid can be synthesized through two distinct routes: the phenylalanine ammonia–lyase (PAL) or Phenylpropanoid pathway and the isochorismate (IC) pathway [114] (Figure 5). While the first enzyme of the PAL pathway is absent from most microalgae [110], not much is known about the expression of the IC pathway in those organisms. Both PAL and isochorismate synthase (ICS) genes were missing from green rhizosphere-associated microalgae *Micractinium rhizosphaerae* [115]. This species, however, possesses a PHYLLO protein homolog, a multifunctional protein with the primary role of phylloquinone (vitamin K1) biosynthesis, a vital component of the PSI electron transport chain [115]. One of the functionalities of this protein is also that of isochorismate synthase. Therefore, it is possible that, at least in green microalgae, salicylic acid biosynthesis occurs de novo and is coupled with photosynthesis. In Figure 7, significant factors for the concentration of individual phenolics in dry biomass are presented. Light intensity was not significant to salicylic acid; it was, however, for protocatechuic, and cinnamic acids, as well as other products of the Phenylpropanoid pathway. The question remains how products of the Phenylpropanoid pathway and daughter pathways, such as flavonoids (Figure 6), are present in microalgae lacking the PAL or TAL enzymes.

### 3.3. Biotic and Abiotic Degradation

**Figure 8:**
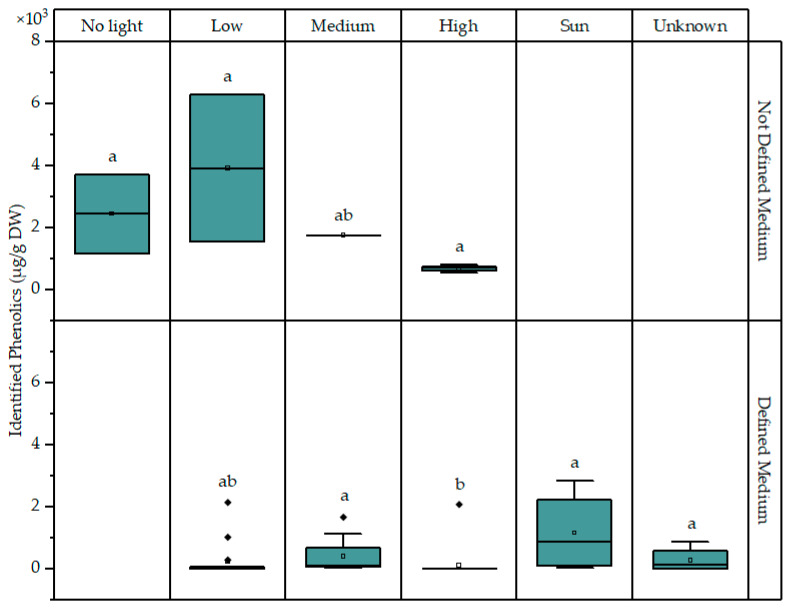
Concentration of total identified phenolics in microalgae cultivated under different light intensities in a defined or undefined medium. Data points corresponding to conditions with copper stress have been excluded to rule out interference. Significant differences (*p* < 0.05) are indicated through compact letter displays. Boxes contain values between the first and third quartiles, while the minimum and maximum values are indicated with horizontal bars at the end of the lines that extend from the boxes. Outliers (marked with rhombi) are considered to be values that are 1.5 times outside the interquartile range (the difference between the first and third quartiles).

A problem with evaluating the effects of light intensity on the concentration of phenolics is that it might affect both the biochemistry of the microalgae and the stability of the measured molecules. For example, caffeic acid is prone to photodegradation, especially in the presence of metal ions, with one of its degradation products being protocatechuic acid [116,117,118]. This could partially explain the results of Sozmen et al., showing a rapid drop in caffeic acid levels with the increase in light intensity [90]. Schnarr et al. reported the abiotic degradation of eight externally supplemented flavonoids in microalgal cultures, with degradation products including hydrobenzoic acids and phloroglucinol [119]. The biodegradation of external phenolics is also generally higher in the presence of light [120]. Microalgae can also utilize phenol as a carbon source. However, functional groups of more complex phenolics present in the aromatic ring need to be removed before complete assimilation is possible [121]. Bisphenols were rapidly metabolized by *Chlamydomonas mexicana*; a limited amount was adsorbed to the cells and no bioaccumulation was detected. However, methylparaben was completely biodegraded in 6 days by *Chorella vulgaris* and, to a lesser extent, by *Phaeodactylum tricornutum*, with possible degradation products including catechol and hydroxybenzoic acids [122]. Figure 8 presents the concentration of the total identified phenolics in microalgae cultivated in defined and undefined growth media under different light intensities. Under a high light intensity (>140 μmol ph m^−2^ s^−1^) the phenolic concentration seems to be lower than under low and moderate light intensities, in both cases. Also, phenolic content was generally higher for the undefined medium, something which will be explored in the next section. In conclusion, changes in the phenolic content and profile of microalgae under varying growth conditions are very difficult to interpret, especially in the presence of light, while in the presence of external phenolics, a range of biotic and abiotic degradation products can occur.

### 3.4. Possible Uptake of Phenolics from Non-Defined Media

Contrary to PAL and TAL, other enzymes of the PP pathway are conserved in the microalgae of most clades [110]. Therefore, if externally provided precursors are available, a wide range of phenolics could be produced. One possible source of external phenolics is non-defined media such as the olive mill waste (OMW) used by Maalej et al. [44]. In that specific case, it is straightforward to attribute the high concentration of hydroxytyrosol and coumaric acid found in the microalgal extracts to the use of the specific substrate, since it is known to be rich in those compounds [123]. Other possible sources of phenolics, however, are not obvious. One example is the use of soil extracts in growth media such as Soil Extract Medium (SEM) [124]. Soil can be rich in simple phenolics such as p-hydroxybenzoic, vanillic, p-coumaric, and ferulic acids [125]. It is unknown how many laboratories use such media in the maintenance of their microalgae, since this information is rarely mentioned. Other non-defined media that might be considered as possible sources of phenolics are growth media containing animal products or derivatives of dairy products. For example, as mentioned before, Sozmen et al. used Bristol medium, which contains the animal product peptone, while Zimermannet al. used whey permeate, a dairy product derivative. Farmed animals consume large amounts of phenols and polyphenols, which can cross most blood barriers, including the very tightly regulated blood–brain barrier [126], while compounds such as cinnamic, ferulic, and p-coumaric acids are present and can even accumulate in muscle and other tissues [127]. Figure S2 shows the total content in identified phenolics of different microalgal phyla cultivated in defined and non-defined media without heavy metal stress. For Chlorophyta, for which the most information is available, the influence of the defined medium is significant. The medium composition is also significant for specific compounds, such as cinnamic and salicylic acids, the flavanone naringenin, and the flavonol kampferol (Figure 6). Therefore, when a non-defined medium is used for microalgal growth, its phenolic content should be examined, if the molecular characterization and quantification of microalgae polyphenols is the goal. 

### 3.5. Accumulation of Phenolics Via Adsorption to the Cell Wall

Even if defined media are used in the main experiments, the accumulated phenolics from the maintenance cultures could eventually influence the main experiment. As mentioned before, it is unknown how many laboratories use soil extracts in their maintenance cultures, while natural seawater, which contains a variety of organic compounds, is often used in the cultivation and maintenance of marine microalgae. Therefore, the absorption or adsorption of external phenolics from maintenance cultures is a potential source of contamination. This becomes evident when the literature on the removal of phenolics from waste streams is considered. *Tetraselmis suecica* removed 85–100% of 50–100 mg/L of phenol, p-cresol and o-cresol, mainly by adsorption [128]. Mollo et al. examined the removal of high concentrations of tyrosol, coumaric acid, and caffeic acid, phenolics found in olive mill waste, via various microalgae [129]. They observed an increase in removal when using species that have a cell wall. *Nannochloropsis* removed almost all the phenolics while cell death was also observed. However, species lacking rigid cell walls (*D. salina* and *I. gralbana*) showed limited phenolic removal compared to the other species. This implies that cell wall adsorption was significant. The rapid adsorption of external phenolics by microalgae also implies that, even at low external concentrations, a significant amount of phenolics could accumulate on the cells. Although the phenolic content of the biomass was not measured in the case of Mollo et al., one can assume it would be especially high, since 23–42 mg/L of the phenolics were almost completely adsorbed to the cells used for the inoculation of the growth medium. It must be noted that the authors used a negative control without a biomass; thus, abiotic degradation can be neglected. Therefore, even the low concentration of phenolics in seawater reported by López et al. could have a significant impact [93]. Given that natural seawater is often used for cultivation and maintenance, it is vital that future research either is performed with DI water and defined media or that all components of the growth media are characterized according to their phenolic content. 

**Figure 9:**
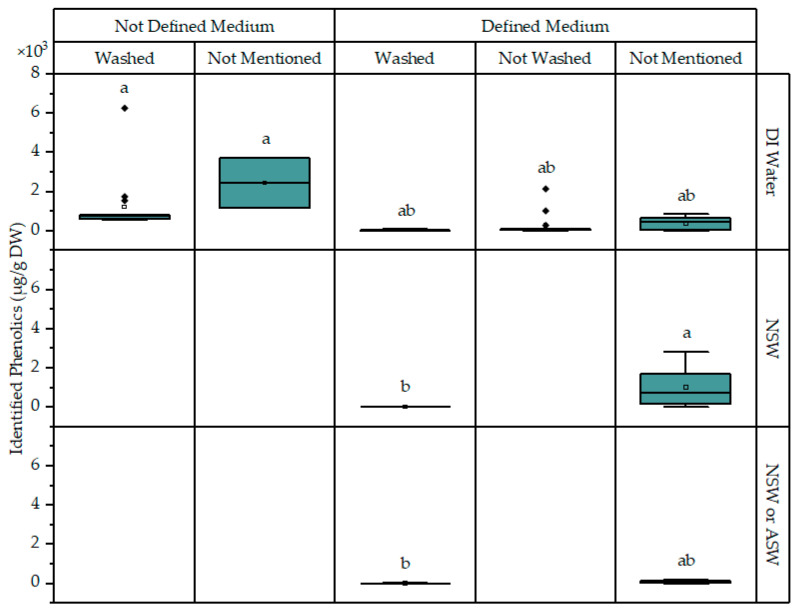
Concentration of total identified phenolics in microalgae cultivated in defined or undefined media based on DI water, natural seawater, or saline water of unknown origin. A further distinction is made between cases where cells were known to be washed or not washed, and cases where this information is unknown. Data points corresponding to conditions with copper stress have been excluded to rule out interference. Significant differences (*p* < 0.05) are indicated with compact letter display. Boxes contain values between the first and third quartiles, while the minimum and maximum values are indicated with horizontal bars at the end of the lines that extend from the boxes. Outliers (marked with rhombi) are considered when values are more than 1.5 times outside the interquartile range (the difference between the first and third quartiles).

Another implication of the adsorption of phenolics on microalgae is the possible effect of washing the cells prior to analyses. This practice is not performed by all labs, with many researchers not explicitly mentioning if it was applied or not. Even when the collected biomass is washed, different approaches to this can be taken, with some groups resuspending the pellet obtained from centrifugation while others simply rinse it with the washing medium. Figure 9 presents the phenolic content of microalgae cultivated in medium based on DI water or natural seawater; the cases where it is not clear if artificial seawater or natural seawater was used are pooled. Washed cells had a lower phenolic content, especially in cases where natural seawater was used; natural seawater seems to have a positive influence on the phenolic content. Also, for defined media, the use of natural seawater resulted in a higher, but not statistically significantly different, phenolic content in the case when washing was not applied or washing was not specified. Cell washing was also significant for several individual phenolics, like gallic and protocatechuic acids, coumaric acid, and epicatechin (Figure 7). 

From the above, it can be concluded that the presence of external phenolics potentially influences the measured phenolic content of microalgae at various steps of the experimental process, spanning from the maintenance conditions to the treatment of the biomass after harvesting. 

## 4. Discussion

While the reactivity of many different non-phenolic compounds towards the Folin–Ciocalteu reagent has been known for a long time, this method is still extensively used. In plant or food sciences, where rich sources of phenolic compounds are often examined, the contribution of these interferences might be small, and corrective modifications to the assay have been developed. Microalgae, however, should not be treated as plant samples. They contain 2–3 times more pigment than plant leaves [50,80,130], as well as much higher protein and nucleic acid contents, which are all reactive towards the FC reagent. Therefore, the use of an FC assay should be either abandoned in microalgal research or FC assays should be used with precautions; for example, FC assays should only be used for specific solvents, with adequate post-treatment methods. Unfortunately, the chromatographic estimation of phenolic content is also prone to systematic errors, as previously discussed. When the research goal is the characterization of phenolic compounds, each step in the experimental process, from the maintenance of the microorganisms to the final cultivation medium, should be free of such molecules. Additionally, all treatments, such as determining the level of applied light intensity, cell washing, and post-treatment of the extract should be accurately reported. Ideally, extract yields should also be reported, while the unit of the concentration that is being presented should be clearly stated. A point that must be made is that a large portion, if not the majority, of the published research does not explicitly mention if the presented results are being presented in terms of dry biomass or dry extract. This was also addressed by Torres et al., who recently reviewed the use of the FC assay in the estimation of the phenolic content of macroalgae and green microalgae [11]. They also point out that 98.7% of articles do not consider interfering compounds. 

Apart from the obvious problems in the methodology used for the study of phenolics in microalgae, another issue that arises is its practical necessity. This research subject has long left the realms of botany and marine biology and entered that of applied science, which aims to develop sustainable and environmentally friendly commodities and services. Even if small amounts of phenolic acids or flavonoids are produced de novo by specific microalgae, the same compounds can be found in large quantities in the waste products of plant matter processing, such as olive mill waste, grape and tomato processing, etc. A number of studies have specifically focused on the remediation of OMW with microalgae, due to the high content of phenolics such as tyrosol, hydroxytyrosol, oleuropein, catechol, vanillic acid, p-coumaric acid, caffeic acid, elenolic acid, verbascoside, and rutin present in this waste stream [120]. In that sense, efforts to increase phenolic production by microalgae via strain selection or genetic engineering are pointless. On the contrary, well-known microalgal metabolites such as essential amino acids-complete vegan proteins, pigments, and fatty acids are not found in plants or other sustainable sources in adequate amounts but are already produced by microalgae in large quantities, while further optimization is possible. The utilization of phenolics by microalgae as biostimulants and/or carbon sources is particularly interesting, since it simultaneously allows for enhanced growth and the removal of those compounds from waste streams. The use of OMW, tomato processing waste, or similar “clean” waste streams has the additional benefit that these sources can be used for food production, a growing application of microalgae. Additionally, the transformation products of phenolics like conjugates with sugars or peptides might have interesting bioactivities, and further research on this is required. Industrial waste containing phenols, bisphenols, or similar molecules can be used to produce biofuel, bioplastics, or other non-food/feed/pharma-related products. Lastly, the analysis showed that copper stress might be one of the few true stimulants of de novo phenolic synthesis in diatoms. Combined with the literature on the bioremediation of phenolics from microalgae, this evidence suggests the notable potential of those organisms in waste treatment.

## 5. Methods

### 5.1. Data Collection

For the present analysis, most published data on phenolic contents were collected and evaluated. The search first used Scopus (https://www.scopus.com/home.uri, accessed on 15 January 2024), with the keywords “microalgae” and “phenolic”. Google Scholar (https://scholar.google.com/, accessed on 18 January 2024) was also used, with a narrower search, including the search terms “Folin” or “chromatography” or “hplc” or “lc-ms”. There were no limits regarding the date of publication, while the literature research started in January 2024 and ended on the 30th of June, 2024. The main aim was to identify problems in the current literature on microalgal phenolics and not to perform a full review of this research area, so some relevant references may have been neglected. The results were screened for their relevance, since a large number did not contain data on phenolic content. References that passed the screening process were further evaluated for the usability of the presented data. The criteria for exclusion were either a lack of clarity in the presented results or use of non-standard units; for example, Quercetin equivalents instead of Gallic acid equivalents (for the FC assay). Data were collected and separated in five different data sets, two regarding the TPC of dry biomass (DW) and dry extract (DE), one regarding the mass ratio of gallic acid equivalents (GAE) to chlorophyll, one containing qualitative chromatographic data, and one containing quantitative chromatographic data. The last data set contained some entries that were provided in non-standard units. A total of 39 references were used for the phenolic content in terms of dry biomass weight, and 36 references were used for phenolic content in terms of dry extract weight. The number of references containing quantitative and qualitative chromatographic data was twenty-one and seven, respectively. In total, 17 references were used to calculate the gallic acid equivalent-to-chlorophyll ratio. In specific cases, conversion to standard units (μg/g DW) was trivial; in others, however, assumptions needed to be made, which are presented in the next section. For the data set showing the mass ratio of gallic acid equivalents (GAE) to chlorophyll, data that were otherwise non-usable, like grams or mols per cell, were included if the mass ratio of GAE:Chl could be calculated. Another type of data that was not usable was TPC in terms of DE when a supplementary extraction yield was not presented.

### 5.2. Data Treatment

The units considered as standard and used in this work were mg GAE/g DW or mg GAE/g DE (dry extract) for TPC data and μg/g DW or μg/g DE for chromatographic data. Individual phenolic concentrations provided in mol per volume or mass were converted to grams using the molar mass. In two specific cases where the phenolic content was provided in mol/cell, the average cell weight was used to estimate the phenolic content in terms of dry weight. The first case was that of *Phaeodactylum tricornutum* in Rico et al., 2013, and Santiago-Díaz et al., 2023 [99,103], where an average dry cell mass of ~8 × 10^−8^ mg/cell was used [131,132]. The second was that of *Dunaliella tertiolecta* in López et al., 2015 [93], where an average dry cell mass of ~6 × 10^−7^ mg/cell was used [133,134].

### 5.3. Estimation of Chlorophyll Interference

A rough estimate of ~3.5% Chl in dry microalgal biomass under nutrient-replete and not-stressful conditions was based on the literature sources [40,50,64,135] as well as unpublished data. A response of chlorophyll to FC of ~0.1–0.88 GAE:Chl w:w was estimated based on the findings of Andriopoulos et al. and Ben Hamouda et al. [39,40]. The data and assumptions used for the calculations are provided in the Supplementary Materials (Limits for chlorophyll interference? Tables S1 and S2). Regarding the lower limit, it was calculated using the assumption that chlorophyll was solely responsible for the TPC observed in methanolic extracts by Andriopoulos et al., since no peaks of an aromatic nature were detected in those extracts. Therefore, the TPC (mg GAE/g DW) was divided by the chlorophyll content (mg Chl/g DW) to yield an average of 0.15 ± 0.03 mg GAE/mg Chl. The upper limit was calculated from the data of Ben Hamouda et al. The authors measured the TPC of ethanolic extract (95%) before and after the removal of pigments. They also asserted that only chlorophyll, not carotenoids, interferes with the FC assay. Therefore, the difference between the TPC before and after the removal of pigments (pg GAE/cell) was divided by the total chlorophyll content (pg/cell) to yield ~0.88 pg GAE/pg Chl.

### 5.4. Statistical Analysis

Standard ANOVA was not suitable in any case, since residuals were not normally distributed. In this context, residuals are defined as the differences between the data-points of a group and the group mean. Instead, the Kruskal–Wallis test (*p* < 0.05) was used to compare different groups, with the Bonferroni-Dunn test applied in all cases where compact letter display is used. In the case of Figure 7 the Benjamini-Hochberg procedure [136] was used to assess statistical significance instead of the Bonferroni correction, since the latter is prone to Type II (false negative) errors when the number of hypotheses is large. The Bonferroni correction should not be confused with the Bonferroni-Dunn test, with the former being a correction for the significance level, while the latter being a post-hoc analysis method. All analyses were performed in Matlab, while the Benjamini-Hochberg procedure was performed manually in Excel spreadsheets. Epsilon squared was calculated according to Tomczak and Tomczak [137], with the data and calculations provided in Supplementary Data—Epsilon SQ calculation.

## 6. Conclusions

This review highlighted significant challenges in accurately determining the phenolic content of eukaryotic microalgae and has underscored the need for more robust and precise experimental methods. The Folin–Ciocalteu (FC) assay, while useful in plant sciences, is unsuitable for microalgal research due to the high presence of interfering compounds such as chlorophyll, proteins, and nucleic acids in microalgal extracts. Despite chromatographic evidence indicating the presence of simple phenolic acids in various microalgae, the absence of critical phenolic biosynthesis enzymes and the variability in their phenolic profiles necessitate further comprehensive research. Moreover, the potential uptake of external phenolics by microalgae, as well as the abiotic degradation of phenolics, have been overlooked, making the interpretation of the current literature findings very difficult. However, the potential for microalgae to absorb, internalize, and transform phenolic compounds from waste streams is well documented. Future research should explore the utilization of external phenolics by microalgae and their complex biotransformation products, as well as their role in heavy metal bioremediation and waste treatment, using adequate and well documented methods.

## Data Availability

All data directly used to derive the results presented in this article are available in the Supplementary Materials. Additional data can be made available upon request.

## Supplementary Materials

The following supporting information can be downloaded at: https://www.mdpi.com/article/10.3390/md22100460/s1, Supplementary Figures.docx, Supplementary Data.xls, Table S1.xls, Limits for chlorophyll interference.docx. Figure S1 in Supplementary Figures.docx. References used for the calculation of the ratio of gallic acid equivalents (GAE) to chlorophyll. The dotted vertical line indicates the interference observed by Ben Hamouda et al., ~0.88 GAE:chl w:w. Significant differences (*p* < 0.05) are indicated with compact letter display. Boxes contain values between the first and third quartiles, while the minimum and maximum values are indicated with vertical bars at the end of lines that extend from the boxes. Outliers are considered when values are more than 1.5 times outside the interquartile range (the difference between the first and third quartiles). Figure S2 in Supplementary Figures.docx. Total identified phenolics in different microalgal phyla cultivated in defined or not defined medium in the absence or presence of copper stress. Significant differences (*p* < 0.05) are indicated with compact letter display. Boxes contain values between the first and third quartiles, while the minimum and maximum values are indicated with vertical bars at the end of lines that extend from the boxes. Outliers are considered when values are more than 1.5 times outside the interquartile range (the difference between the first and third quartiles). Table S1 in Table S1.xls. Phenolic compounds that have been studied in microalgal samples of different phyla, from left to right Chlorophyta, Dinoflagellata, Cryptophyta, Euglenophyta, Haptophyta, Heterokontophyta, and Rhodophyta. Plus, minus and plus-minus signs indicate positive, negative, and positve and negative detection respectively. The number of positive or negative detections are given for each molecule (bars on the right of the table), while the number of references for each genus are also mentioned (bar at the bottom of the table). Table S2 in Limits for chlorophyll interference.docx. Data for the estimation of the lower limit for chlorophyll interference (Andriopoulos et al., 2022a [40]). Table S3 in Limits for chlorophyll interference.docx. Data for the estimation of the lower limit for chlorophyll interference (Ben Hamouda et al., 2022 [39]).
