# Supplementary material for: Microalgal Phenolics: Systematic Review with a Focus on Methodological Assessment and Meta-Analysis
Source: Mar Drugs. 2024 Oct 7;22(10):460. doi: 10.3390/md22100460 (PMC11509163; doi:10.3390/md22100460)
Supplement: Supplementary file 1 [file marinedrugs-22-00460-s001.zip › Limits for chlorophyll interference.pdf]

Table S2. Data for the estimation of the lower limit for chlorophyll interference (Andriopoulos et al., 2022a [40]).

Species	*Chlorella minutissima*	*Dunaliella salina*	*Nannochloropsis oculata*	*Tisochrysis lutea*	*Isochrysis galbana*
Chla % DW *	2.8	0.88	0.71	0.95	1.01
Chla (mg/g DW)	28	8.8	7.1	9.5	10.1
Chlb % DW	1.01	0.29	0	0	0.08
Chlb (mg/g DW)	10.1	2.9	0	0	0.8
Sum Chl (mg/g DW)	38.1	11.7	7.1	9.5	10.9
TPC (mg GAE/g DW)**	6.23	1.3	1.3	1.25	1.78
GAE:Chl (w:w) ***	0.16	0.11	0.18	0.13	0.16
Average	0.15				
sd	0.03				

* Chlorophyll measured in biomass

** TPC of methanolic extracts

*** Assuming that all pigments were extracted

Table S3. Data for the estimation of the lower limit for chlorophyll interference (Ben Hamouda et al., 2022 [39]).

Chla (pg/cell) *	7.39
Chlb (pg/cell)	4.82
Sum Chl (pg/cell)	12.2
TPC (pg GAE/cell) before pigment extraction ** ***	20.63
TPC (pg GAE/cell) after pigment extraction	9.91
GAE:Chl (w:w) ****	0.88

* Measured in MeOH

** Data extracted from figure

*** in 95% EtOH

**** Assuming that all pigments were extracted
