# Supplementary material for: Microalgal Phenolics: Systematic Review with a Focus on Methodological Assessment and Meta-Analysis
Source: Mar Drugs. 2024 Oct 7;22(10):460. doi: 10.3390/md22100460 (PMC11509163; doi:10.3390/md22100460)
Supplement: Supplementary file 1 [file marinedrugs-22-00460-s001.zip › Supplementary Figures.pdf]

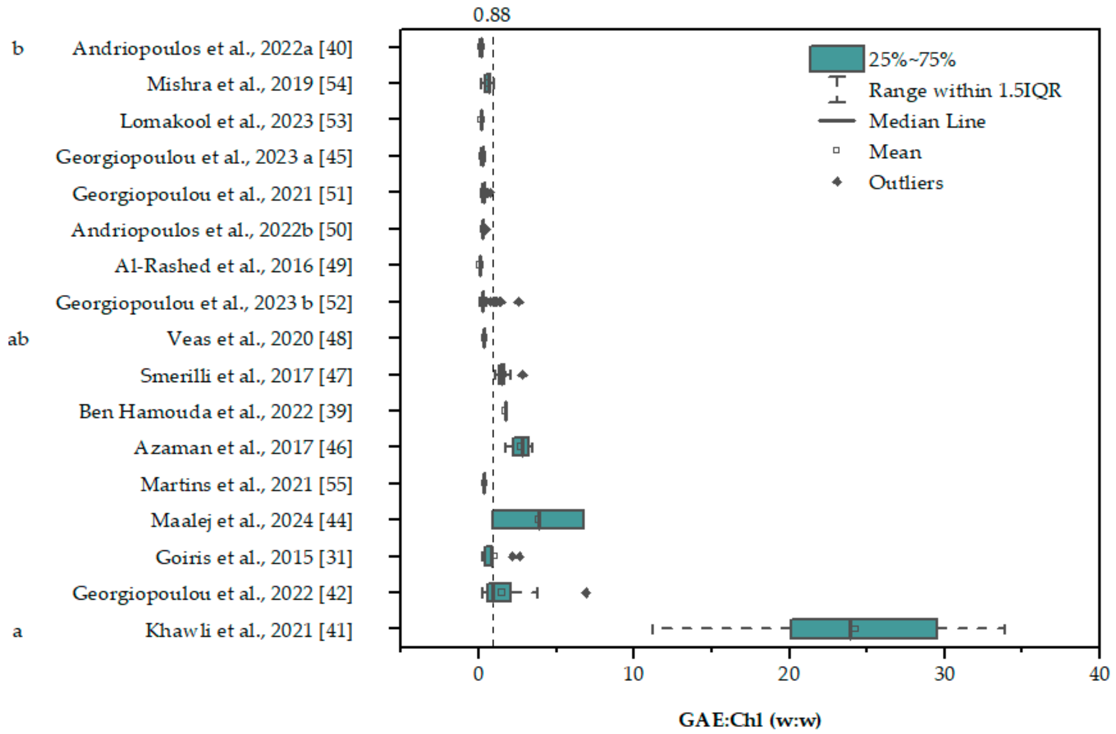

Figure S1. References used for the calculation of the ratio of gallic acid equivalents (GAE) to chlorophyll. The dotted vertical line indicates the interference observed by Ben Hamouda et al., ~ 0.88 GAE:chl w:w. Significant differences ($p < 0.05$) are indicated with compact letter display. Boxes contain values between the first and third quartiles, while the minimum and maximum values are indicated with vertical bars at the end of lines that extend from the boxes. Outliers are considered when values are more than 1.5 times outside the interquartile range (the difference between the first and third quartiles).

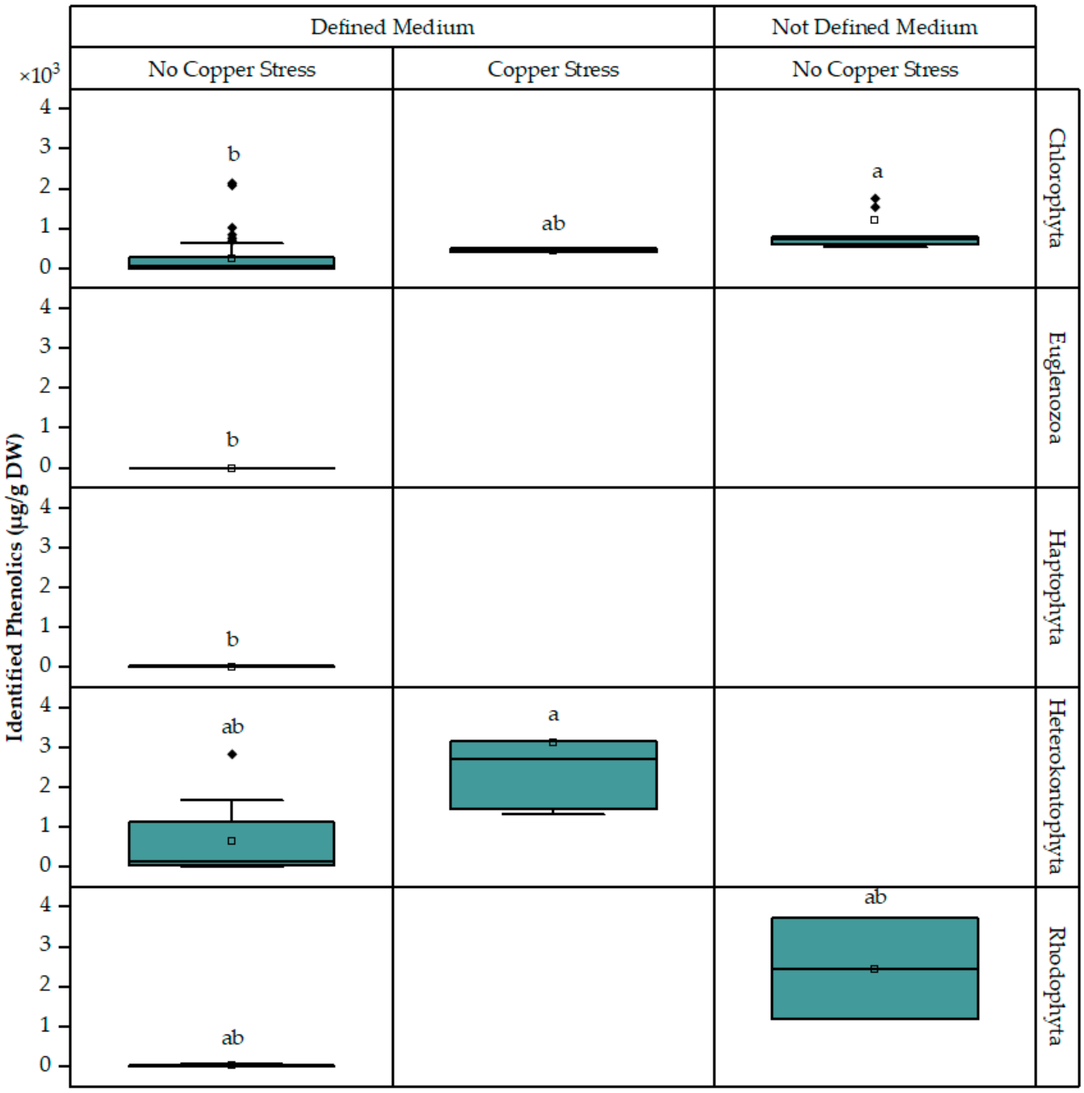

Figure S2. Total identified phenolics in different microalgal phyla cultivated in defined or not defined medium in the absence or presence of copper stress. Significant differences ($p < 0.05$) are indicated with compact letter display. Boxes contain values between the first and third quartiles, while the minimum and maximum values are indicated with vertical bars at the end of lines that extend from the boxes. Outliers are considered when values are more than 1.5 times outside the interquartile range (the difference between the first and third quartiles).
